# Altered Community Flammability in Florida’s Apalachicola Ravines and Implications for the Persistence of the Endangered Conifer *Torreya taxifolia*

**DOI:** 10.1371/journal.pone.0103933

**Published:** 2014-08-01

**Authors:** John M. Mola, J. Morgan Varner, Erik S. Jules, Tova Spector

**Affiliations:** 1 Department of Biological Sciences, Humboldt State University, Arcata, California, United States of America; 2 Department of Forestry, Forest & Wildlife Research Center, Mississippi State University, Mississippi State, Mississippi, United States of America; 3 Florida Park Service, Department of Environmental Protection, Panama City, Florida, United States of America; 4 Graduate Group in Ecology, University of California Davis, Davis, California, United States of America; DOE Pacific Northwest National Laboratory, United States of America

## Abstract

Plant species and communities often reflect historic fire regimes via ecological and evolutionary responses to recurrent fires. Plant communities of the southeastern USA experience a wide array of fire regimes, perhaps nowhere more marked than the juxtaposition of fire-prone uplands and adjacent mesic ravines along Florida’s Apalachicola River. The ravines contain many endemic and disjunct species, most notably the endangered endemic conifer *Torreya taxifolia*. A rapid decline in *T. taxifolia* over the past 60 years has been associated with widespread replacement by other tree species. To understand the changes accompanying the shift in ravine composition, we compared leaf litter flammability of nine historic and contemporary species. We measured maximum flame height, flame duration, smoldering duration, mass loss, absorptive capacity, and drying rate. Ordination and perMANOVA suggest the nine species segregated into three distinct groups: the fire-impeding *T. taxifolia* and *Taxus floridana*; an intermediate group of three deciduous angiosperms; and a mixed cluster of four flammable species. Results suggest *T. taxifolia* and *T. floridana* were fire-impeding species in these communities, while contemporary dominants burn similarly to the upslope pyric species. The increasing presence of fire-facilitating species may portend a shifting fire regime that further imperils *T. taxifolia* and other rare species in the formerly fire-safe ravines.

## Introduction

Fire is a dominant driver of ecological processes in fire-prone environments worldwide [1]. Flammability of senesced leaves, cones, or sloughed bark [2]–[4] can facilitate the spread and intensity of fires in some plant communities and impede or dampen fire in others [5]–[7]. Flammability has been quantified as a combination of ignitibility of fuel (energy required for combustion), combustibility (intensity, flame height, energy output), sustainability (duration of flaming and smoldering), and consumability [mass loss; 8]. Flammable plants promote conditions favoring fire, resulting in the persistence of plant communities with high fire tolerance [2], while plant communities composed of species intolerant of fire tend to burn with lower flammability and dampen the intensity or spread of fire [4], [5]. Understanding the flammability of individual species can provide insight into community fire regimes [9].

Changes in plant community composition that alter fire regimes can initiate feedbacks that govern ecological processes [1], [10], [11]. For example, widespread shifts from pyrophytic to mesophytic tree species in woodlands and forests of eastern North America (termed “mesophication”; 10) have resulted in a positive feedback cycle where the increases in fire-intolerant, less flammable species reduce the intensity and spread of fires in formerly fire-prone environments. Negative feedbacks can also occur where species with low flammability are replaced by more flammable species, as in transitional Amazonian tropical rainforests [e.g., 12,13]. These shifts in fuels may have extreme consequences for fire regimes, affecting ecosystem processes broadly and species of conservation concern more specifically.

**Figure 1:**
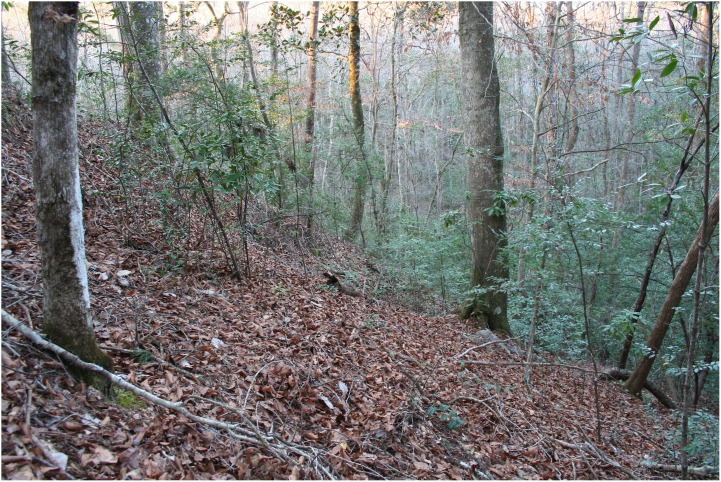
The mesic ravines along the Apalachicola River in northern Florida harbor a variety of rare plant and animal species. 20^th^ century declines in the formerly dominant endemic conifer Torreya taxifolia have resulted in shifting tree community composition.

In this paper, we explore the possibility that the distribution and recovery of a rare tree, *Torreya taxifolia*, is further imperiled due to shifts in the woody plant community and their litter fuels. *Torreya taxifolia* is a federally endangered conifer that has undergone a precipitous decline in the numbers and health of remnant populations [14], [15]. A 35 km corridor along Florida’s Apalachicola River [14] harbors ravines that shelter mesophytic forests and a rich diversity of endemic and disjunct plant species as many taxa found refuge in the cool, moist ravines during the warming interglacial period [Fig. 1; 16]. These ravines hold the only wild populations of the rare conifers *T. taxifolia* and *Taxus floridana* [17]. As recently as the 1950s, *T. taxifolia* comprised ∼15% of the dominant trees within the ravines totaling ca. 300,000 to 650,000 individuals [18], [19]. The current population includes fewer than 2,000 small trees, with few reproductive individuals observed since 1962, representing <1% of the trees in the ravines [14], [20]. Mechanisms proposed to explain the decline of *T. taxifolia* include fungal pathogens, water stress, regional warming, hydrologic changes, and altered fire regimes [21], [22]. While the roles of pathogens, water stress, and warming climate have been addressed in previous work [21], [22], the role of fire in the decline and potential recovery of *T. taxifolia* has not been studied.

It remains unknown how fire affected the decline, or may affect the future recovery, of *T. taxifolia*. It has been proposed that fire frequency of the steephead ravines has increased, thereby making it difficult for the thin-barked, fire-susceptible *T. taxifolia* to recruit new individuals [15], [21]. It has also been proposed that smoke from historic fires in the adjacent uplands helped to suppress fungal loads that currently plague remnant trees in the ravines [21]. The Apalachicola ravines have been described as some of the most “fire-proof” areas of Florida [18], [23], while others contend that fire-caused canopy gaps may have helped sustain *T. taxifolia* prior to fire exclusion [15], [21]. In spite of this disagreement, no studies have been conducted on the flammability of *T. taxifolia* or its associated species. Anecdotal evidence suggests fire spreads into the ravines during upslope prescribed burns and has been observed to creep through the ravines for several days (J. McKenzie, personal communication; D. Prentiss, personal communication). These fires may result in the eventual resprouting or mortality of *T. taxifolia* [24; E. Johnson, personal communication]. It is unclear, however, if these ravine fires are consistent with historical patterns, or if they are novel due to the changing species and resulting fuel composition of the ravines. The lack of studies on fire in the ravines is especially noteworthy given the extensive attention given the role of fire and flammability in the pine-oak ecosystems immediately upslope [e.g., 5,6,25,26].

If fire played a dominant role in the ravines historically, a necessary condition would have been the presence of flammable fuels. Following the logic of Mutch [2], we proposed that if the flammability of historic ravine species is high, then fire may have played a persistent role in ravine plant dynamics. If, however, contemporary fuels are more flammable than historic fuels in *T. taxifolia* –dominated communities, then this shift would suggest negative fire feedbacks, as described for wet Neotropical forests [12], [13], [27]. To better understand the role of fire in ravine communities past and present, we evaluated the flammability of ravine species, comparing historic and contemporary tree community composition. Results will help clarify the role and potential effects of fire in the ravine ecosystems and, more broadly, help shed light on the effects of shifting species composition on fire regimes.

## Methods

We collected surface litter from nine currently or formerly dominant tree species in the Apalachicola ravines. We collected eight species from Torreya State Park, Florida: *T. taxifolia*, *T. floridana, Liquidambar styraciflua, Liriodendron tulipifera, Ilex opaca, Magnolia grandiflora, Fagus grandifolia,* and *Ostrya virginiana*. Due to its rarity, *T. taxifolia* litter was also collected at Maclay Gardens State Park and Three Rivers State Park, Florida. We also used previously collected *Pinus glabra* litter collected from the Tuskegee National Forest, Alabama, though the species is found in the Apalachicola ravines. Litter from all species was collected in May and June, during the typical fire season in the region [28]. For each species, we collected ∼200 g of surface litter (dry weight; ∼20 g from ten separate trees of each of the nine species). Access and permits for collecting litter were provided by the Florida Division of Recreation and Parks. Samples were transported to the laboratory with less than seven days between field collection and lab preparation.

**Figure 2:**
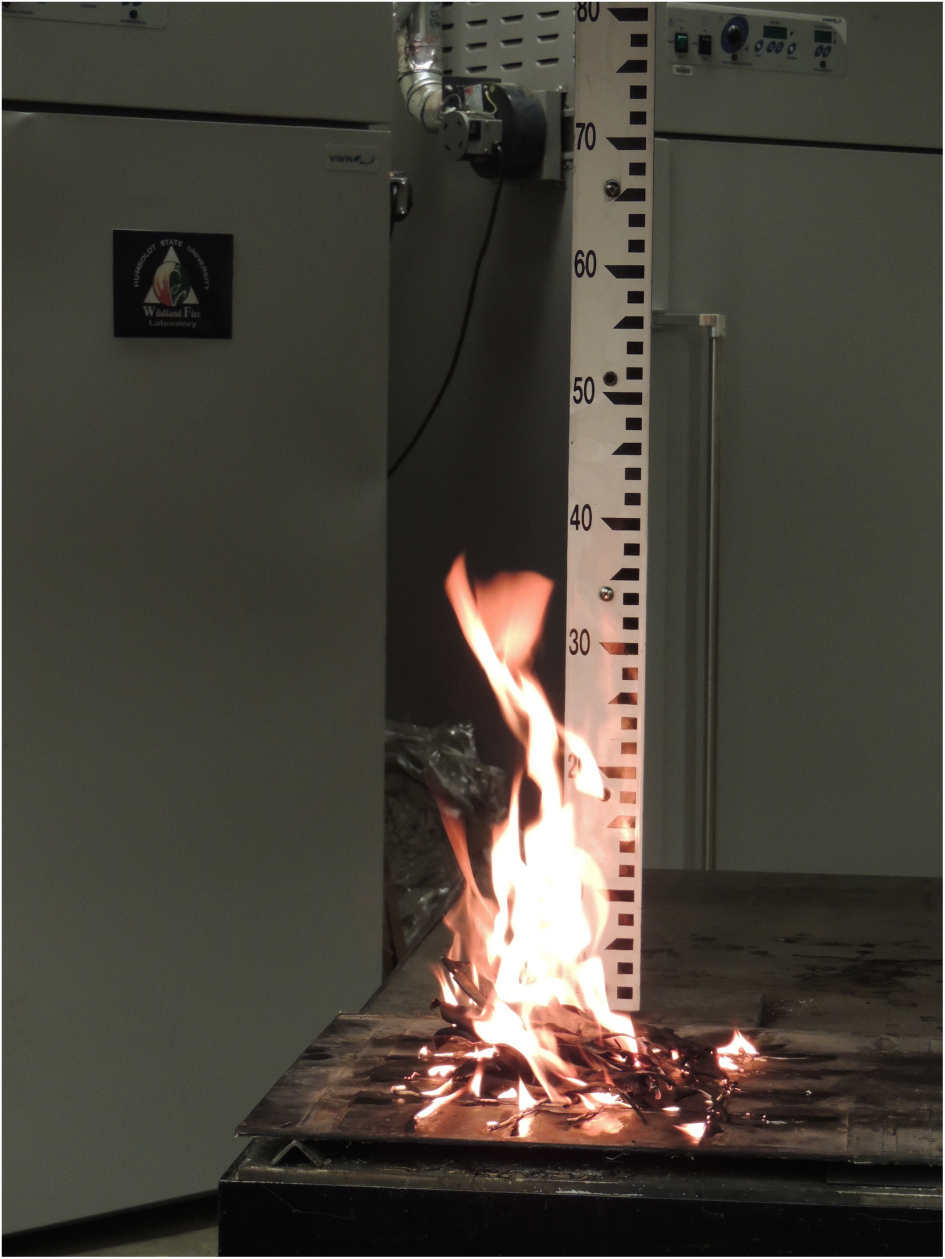
A laboratory flammability experiment with *Magnolia grandiflora*, a native tree species in Florida’s Apalachicola ravines. *M. grandiflora* is one of several tree species that have increased following the demise of the endemic conifer *Torreya taxifolia*. The resulting shift in species composition has cascading effects on community flammability and consequences for the recovery of *Torreya taxifolia* and perhaps a wider suite of fire-sensitive ravine taxa.

In the lab, litter was sorted to remove other species and any woody debris. Five to seven replicates of each of the nine species were oven-dried at 70°C for 48 hours. After drying, 15±0.1 g (±0.5 g for the large-leaved *M. grandiflora*) of litter were burned using standard methods [3], [5]. We distributed 15 g of litter across the inner 20×20 cm area of a 35×35 cm grid of eight xylene-soaked cotton strings on a stainless steel platform beneath a 2.75×2.75 m ventilation hood with constant draw. Strings were ignited and a timer started once the litter ignited. Flame height was visually estimated using a 125 cm ruler (with 1 cm graduations) behind the fuel (Fig. 2). Timers captured the point when flames extinguished (flaming time, sec). The duration of smoldering (sec) was measured until glowing stopped. The remaining ash and unburned litter were collected from the platform and weighed to calculate mass loss (%).

We coupled our burning data with data from litter drying experiments on the same nine species from our sites [7; and our own unpublished data; See Appendix S1 for full data]. In humid, punctuated wet-dry climates like those in northern Florida [23], “dry flammability” measures such as ours can be misleading, since oven-dry conditions are rare on landscapes that receive abundant or frequent precipitation and have otherwise high relative humidity. To account for these factors, we used two additional metrics of litter flammability: drying time [hours, as measured by time lag; 7] and their initial moisture content (%) following prolonged saturation [or absorptive capacity; 7]. Detailed methods are presented elsewhere [7] but entailed saturating four 15 g litter samples for 24 hours, measuring their water absorption (following agitation to remove excess water), and tracking their moisture loss over seven days in a constant drying environment (air temperature 23° to 24°C, 45 to 50% relative humidity). We used data for five species from Kreye et al. [7] and unpublished data for *T. taxifolia*, *T. floridana*, *O. virginiana*, and *P. glabra* measured in the same experiments.

Because our burning and drying response variables were highly correlated, we used principal components analysis (PCA) to assess differences in multiple flammability measures among our nine taxa. We included the four burning metrics (flame height, flame duration, smoldering time, and mass loss) and the two drying metrics (time lag and initial moisture content) in our PCA, and we relativized each variable based on the number of standard deviations from the mean. To determine if species could be segregated into discrete groups based on the burning and drying characteristics, we used *k*-means cluster analysis. Next, we tested for differences in burning and drying characteristics among the groups identified in the cluster analysis using perMANOVA. We used the non-parametric perMANOVA because we could not meet the assumptions of normality of MANOVA. The PCA and cluster analysis were performed with NCSS [29] and the perMANOVA was done using the adonis function in the vegan package for R version 3.01 [30].

## Results

Litter from the nine ravine tree species differed in several aspects of flammability (Table 1). Mean maximum flame height differed among the nine species burned. *Liriodendron tulipifera* and *F. grandifolia* burned with the tallest flames with means of 84.6 cm and 81 cm, respectively. *Torreya taxifolia* had the lowest mean flame height at only 26.2 cm. Fuel consumption for the nine species segregated into two distinct groups with most species having means ranging from 73 to 89% consumption and *T. taxifolia* and *T. floridana* being different from all others with only 44% and 41% mass loss, respectively. *Torreya taxifolia* and *T. floridana* had the longest duration flaming at 155 and 145 sec, respectively. *Pinus glabra* had the longest smoldering duration at 278 sec and *T. taxifolia* had the shortest duration at 128 sec.

**Table 1:** Litter flammability of ravine and upland tree species, sorted by maximum flame height.

Species	Community	Flameheight(cm)	Flameduration(sec)	Massloss(%)	Smolderingtime (s)	Dryingtime (hr)	Initial moisturecontent (g)
*Liriodendron tulipifera*	Ravine	84.6	48.6	0.89	173.4	4.81	386.83
*Quercus laevis* a	Upland	81.4	50.4	0.90	350.7	-	-
*Fagus grandifolia*	Ravine	81.0	50.8	0.86	177.6	6.86	414.67
*Pinus glabra*	Ravine	78.3	53.1	0.88	278.7	4.95	127.33
*Magnolia grandiflora*	Ravine	76.4	55.2	0.87	123.2	6.42	226.50
*Quercus falcata* a	Upland	75.0	52.0	0.87	350.7	-	-
*Ilex opaca*	Ravine	68.6	74.4	0.89	144.8	7.30	188.50
*Quercus stellata* a	Upland	68.4	52.1	0.85	216.3	-	-
*Quercus margaretta* a	Upland	68.1	66.1	0.79	286.5	-	-
*Quercus nigra* a	Ravine	57.3	77.1	0.78	268.8	-	-
*Liquidambar styraciflua*	Ravine	54.4	48.2	0.87	288.6	2.67	217.17
*Quercus incana* a	Upland	52.1	77.3	0.86	341.8	-	-
*Taxus floridana*	Ravine	46.0	149.4	0.42	267.4	12.33	309.33
*Ostrya virginiana*	Ravine	43.2	69.8	0.74	278.4	8.08	466.33
*Quercus hemisphaerica* a	Ravine	40.6	91.4	0.61	348.0	-	-
*Quercus virginiana* a	Upland	33.6	84.5	0.70	230.7	-	-
*Torreya taxifolia*	Ravine	26.2	155.4	0.44	128.2	9.59	248.00

a Moisture metrics for the ravine species were collected in part from Kreye et al. (2013), moisture metrics for upland species were not collected as part of the Kane et al. (2008) study and so are unavailable.

b Ravine species’ flammability data were collected as part of this study, upland species flammability data are from Kane et al. (2008).

**Figure 3:**
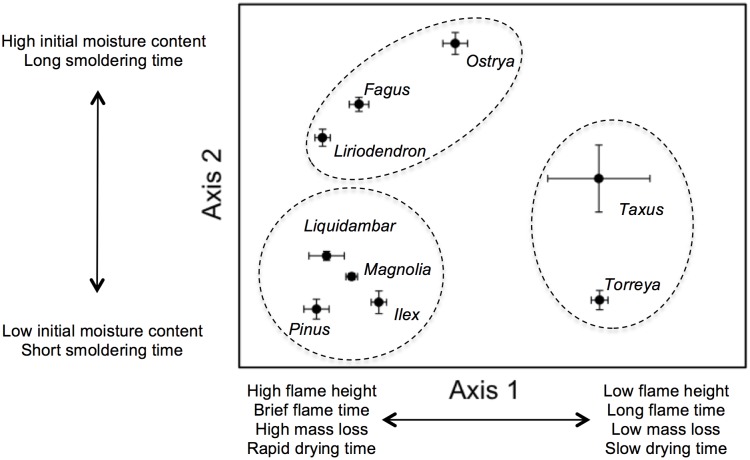
Results of principal components analysis using burning characteristics and drying characteristics for litter of nine woody plant taxa found in the Apalachicola ravines, Florida. The average PCA score (±1.0 S.E.) is shown for each taxa. Circles around taxa are groupings determined using *k*-means clustering analysis.

Combining the four burning and two drying flammability metrics in the PCA explained 54%, 18%, and 16% of the variance for the first, second, and third axes, respectively (88% cumulative). Four flammability metrics were strongly correlated with the first axis (Table 2): those taxa with low axis 1 scores burned with tall flames (*r* = −0.832), brief flaming duration (*r* = 0.912), substantial mass loss (*r* = −0.957), and dried rapidly (*r* = 0.864; Fig. 3). There were strong positive correlations between axis 2 and initial litter moisture content (*r* = 0.899) and modest positive correlations with the duration of smoldering (*r* = 0.404). The third axis was strongly and negatively correlated with smoldering duration (*r* = −0.896); *T. taxifolia* and *T. floridana* differed from other taxa with particularly brief smoldering.

**Table 2:** Factor loadings describing the contribution to three principal components (PC) axes of burning characteristics and drying characteristics for litter of nine woody plant taxa collected in the Apalachicola Ravine, Florida.

Factor	PC 1	PC 2	PC 3
Flame Height	−0.834	0.072	0.107
Flame Duration	0.912	−0.157	−0.149
Smolder Duration	−0.181	0.404	−0.896
Mass Loss	−0.957	0.053	0.014
Drying Time	0.864	0.173	0.004
Initial Moisture Content	0.199	0.899	0.367

The *k*-means cluster analysis segregated the species into three distinct groups explaining 38.95% of the variation in the burning and drying characteristics (Fig. 3), and the perMANOVA demonstrated that the groups differed significantly in these characteristics (*F* = 4.312, df = 46, *P* = 0.021; Fig. 3). The rare conifers *T. taxifolia* and *T. floridana* group with short flame heights, brief flaming and smoldering durations, minor fuel consumption, and high moisture retention. In contrast, three deciduous hardwood trees *O. virginiana*, *F. grandifolia*, and *L. tulipifera* burned with tall flames, flamed and smoldered for long durations, and had heavy fuel consumption while losing their high initial moisture rapidly. The remaining species, evergreens *P. glabra*, *I. opaca*, *M. virginiana*, and deciduous *L. styraciflua* grouped with tall flames, long durations of flaming and smoldering, with heavy fuel consumption while taking on little initial moisture and losing it rapidly.

## Discussion

The observed magnitude of interspecific differences in flammability suggests that major changes in community flammability have transpired in Florida’s Apalachicola ravines. With the decline of *T. taxifolia*, a species with very low flammability (Fig. 3, and the relative increases in relatively flammable species, a dramatic shift in community flammability has occurred. Prior to the decline of *T. taxifolia*, it is likely that *T. taxifolia* litter dampened the intensity and extent of surface fires that spread from the flammable pine-oak savannas upslope [31]. Extant *T. taxifolia* contributes little to the litter fuels because it has declined in both abundance and stature, with the average tree height near only 1 m [14, T.S. unpublished manuscript]. As species such as *I. opaca, F. grandifolia,* and *M. grandiflora* have replaced forest area formerly occupied by *T. taxifolia* (Table 3) [19], the flammability and therefore susceptibility of the ravines to fire has increased. The higher flammability of litter of these successor species may promote the spread and increased intensity of fires within the ravines, further suppressing the recovery of *T. taxifolia* in a negative feedback. Similar fire feedbacks have been found in transitional forests of the tropics [12], [13] and savannas in the temperate zone [11] where these feedbacks can act as stabilizing (negative feedback) or destabilizing (positive feedback) forces. In the ravines, the shift from non-flammable to flammable species has apparently resulted in a negative fire feedback that may further destabilize these formerly fire-safe sites.

**Table 3:** Historic and post-decline dominant tree densities, sorted by percent change in density, and their maximum flame height as determined from our study.

Species	Historic density^a^	Post-decline density^b^	% Density change^c^	Max flame height (cm)
*Fagus grandiflora*	15.3%	25.9%	+69.2%	81.0
*Ilex opaca*	13.1%	18.5%	+41.2%	68.6
*Magnolia grandiflora*	35.5%	43.5%	+22.5%	76.4
*Pinus glabra*	20.9%	12.0%	−42.6%	78.3
*Torreya taxifolia*	14.9%	<0.001%	∼−100%	26.2

a From Harper (1914).

b From Schwartz (2000), only trees with a diameter >15 cm are included.

c Caution should be used in interpreting these results due to the different sampling methods.

Not only have more flammable species replaced *T. taxifolia* within the ravines, but also the flammability of replacement species is somewhat comparable to pyric species in adjacent *Pinus palustris* [3] and *Quercus* spp. [5] uplands. These replacement species burned with flame heights comparable to the upland *Quercus* (*Q. laevis*, *Q. falcata*, *Q. margaretta*, and *Q. stellata*) and *Pinus palustris* [3,5; Table 1]. Among increasing species, *L. styraciflua*, *I. opaca*, and *M. grandiflora* also dry rapidly [7], enabling them to burn under the mesic conditions that characterize the Apalachicola ravines. The flammability of these species shows that, given favorable fire weather, the spread of fires in contemporary communities are more probable than with the low-flammability litter that formerly prevailed in the ravines. Inferences from our results may be limited since the relationship examined here is on the flammability of litter and not necessarily related to species abundance *per se*, but such data cannot be obtained for pre-decline ravine forests. Furthermore, previous research demonstrates that often the most flammable species determines the overall flammability of a given fuel mixture [32]. As a result, the replacement of *T. taxifolia* with more flammable species could have a disproportionate role in increasing community flammability. As such, our results highlight the importance of understanding how declines in dominant species can shift community flammability.

The mechanisms for the shifts in community flammability are unclear. Variation among species, even those phylogenetically similar, has been found elsewhere [3], [33], [34]. In several species, burning and drying rates were linked to leaf traits (long leaves that curled) that generated porous, well-ventilated fuels [7], [33], [34]. In other genera, chemical differences (terpene content and identity) drove interspecific differences in burning [35]. The species that have replaced *T. taxifolia* and the similarly flammable *T. floridana* have different leaf morphologies and presumably divergent chemical compositions. Future research should evaluate traits responsible for interspecific differences in flammability in these species and in other ecosystems where species and resulting fire regimes may be changing.

While it is evident that upland fires creeping into the ravines is not the original cause of *T. taxifolia* decline, our results demonstrate that accounting for the increased fire susceptibility of the ravines will be necessary in parallel with reestablishment efforts. If *T. taxifolia* populations can be reestablished in the wild, understanding the flammability of the ravine community will be crucial in order to sustain those populations until the fuels of this ecosystem recover their former fire-suppressing characteristics. The increased prevalence of fire in the ravines may further imperil *T. taxifolia, T. floridana* and other rare ravine taxa (Appendix S2). Some evidence shows that fire spread was impeded in ∼25% of *T. taxifolia*’s range over the past few decades (M. Schwartz, personal communication). In these cases, fire was apparently impeded due to previous upslope management, which included the creation of windrows and the establishment of fire-intolerant species upslope (T. S., personal observations). Upslope and ecotonal areas that were not so heavily altered by such ground disturbance and recent upslope restoration efforts may provide the bridge for recent fires to spread into the ravines (J. McKenzie, personal communication, D. Prentiss, personal communication) and supports our concern that future fires may complicate *T. taxifolia* population recovery.

The Apalachicola ravines are a biodiversity hotspot especially for disjunct and rare flora [36]; ∼48 rare plant species occur in the ravines, of which 41 are considered endangered and 6 threatened in Florida (Appendix S2). Many of the rare taxa found in the ravines depend on the cool, stable temperatures, moist soils, and humid microclimates [16], [31]. In addition to the general fire sensitivity of these taxa, fire regime changes in the ravines could alter the microclimate by opening the canopy, increasing winds and decreasing humidity potentially having cascading effects on the rare ravine taxa.

As individual species decline due to habitat loss, the introduction of invasive species, and the spread of emerging pathogens, examples of the negative feedback phenomenon we describe may become more common. For example, the functional extinction of the flammable tanoak (*Notholithocarpus densiflorus*) in coastal California and Oregon forests [37] or the loss of the non-flammable, foundation tree eastern hemlock (*Tsuga canadensis*) in eastern US forests [38] may portend shifts in flammability and future fire regimes for remnant communities. Understanding the nuances of individual species interactions with ecosystem processes will be important; replacing species may result in negative, positive, or neutral feedbacks that have the potential to have cascading effects on surviving individuals or on other remnant species [37]. As ecological changes transpire in other ecosystems [39], our task will be to determine where potential fire regime shifts may occur and how, or if, we can intervene.

## Supporting Information

Appendix S1 Data used for analysis.
(CSV)

Appendix S2 Rare plant taxa found in the steephead ravines of the Apalachicola River watershed. Conservation status is shown for each taxa (E = Endangered, T = Threatened). FNAI rank refers to the ranking system used by Florida Natural Areas Inventory (FNAI 2010).
(DOCX)
