# Supplementary material for: Altered Community Flammability in Florida’s Apalachicola Ravines and Implications for the Persistence of the Endangered Conifer Torreya taxifolia
Source: PLoS One. 2014 Aug 1;9(8):e103933. doi: 10.1371/journal.pone.0103933 (PMC4118970; doi:10.1371/journal.pone.0103933)
Supplement: Appendix S2 — Rare plant taxa found in the steephead ravines of the Apalachicola River watershed. Conservation status is shown for each taxa (E = Endangered, T = Threatened). FNAI rank refers to the ranking system used by Florida Natural Areas Inventory (FNAI 2010). (DOCX) [file pone.0103933.s002.docx]

Appendix S2. Rare plant taxa found in the steephead ravines of the Apalachicola River watershed. Conservation status is shown for each taxa (E = Endangered, T = Threatened). FNAI rank refers to the ranking system used by Florida Natural Areas Inventory (FNAI 2010).

| **Species** | **Endemic** | **Florida Status** | **Federal Status** | **FNAI Rank** | **Notes on Fire Regime** |
| --- | --- | --- | --- | --- | --- |
| *Actaea pachypoda* |  | E |  | G5, S1 |  |
| *Anenome americana* |  | E |  | G5, S2 |  |
| *Aquilegia canadensis var. australis* |  | E |  | G5, T1, Q, S1 |  |
| *Asarum arifolium* |  | T |  | G5, S3 |  |
| *Baptisia megacarpa* |  | E |  | G2, S1 |  |
| *Calycanthus floridus* |  | E |  | G5, S2 |  |
| *Carex batzellii* |  | T |  | G3, S3 |  |
| *Cornus alternifolia* |  | E |  | G5, S2 |  |
| *Cryptotaenia canadensis* |  | E |  | G5, S1 |  |
| *Cynoglossum virginianum* |  | E |  | G5, S2 |  |
| *Dirca palustris* |  | E |  | G4, S2 |  |
| *Epigaea repens* |  | E |  | G5, S2 |  |
| *Erythronium umbilicatum* |  | E |  | G5, S2 |  |
| *Euonymus atropurpureus* |  | E |  | G5, S2 |  |
| *Euphorbia commutata* |  | E |  | G5, S2 |  |
| *Forestiera godfreyi* |  | E |  | G2, S2 |  |
| *Goodyera pubescens* |  | E |  | G5, S1 |  |
| *Hexastylis arifolia* |  | T |  | G5, S1 |  |
| *Hybanthus concolor* |  | E |  |  |  |
| *Hydrangea arborescens* |  | E |  | G5, S1 |  |
| *Kalmia latifolia* |  | T |  | G5, S3 |  |
| *Liatris gholsoni* | X | E |  | G1, S1 |  |
| *Lilium michauxii* |  | E |  | G4, G5, S2 |  |
| *Magnolia ashei* |  | E |  | G2, S2 | Fire not recommended (Chafin 2000) |
| *Magnolia pyramidata* |  | E |  | G4, S3 | Fire not recommended (Chafin 2000) |
| *Malaxis unifolia* |  | E |  | G5, S3 |  |
| *Matelea alabamensis* |  | E |  | G2, S2 | Found in upper ravine ecotone with sandhill, fire recommended (Chafin 2000) |
| *Matalea baldwyniana* |  | E |  | G3, S1 |  |
| *Matela floridana* |  | E |  | G2, S2 |  |
| *Medeola virginiana* |  | E |  | G5, S1 |  |
| *Pachysandra procumbens* |  | E |  | G4,G5, S1 |  |
| *Pinckneya bracteata* |  | T |  |  |  |
| *Rhododendron austrinum* |  | E |  | G3, S3 | Fire not recommended (Chafin 2000) |
| *Schisandra glabra* |  | E |  | G3, S2 |  |
| *Sideroxylon lycoides* |  | E |  | G5, S2 |  |
| *Silene polypetala* |  | E | E | G2, S1 | Maintain intact canopy, leaf litter (Chafin 2000, USFWS 1996) |
| *Staphylea trifolia* |  | E |  | G5, S1 |  |
| *Stewartia malacodendron* |  | E |  | G4, S3 |  |
| *Taxus floridana* | X | E |  | G2, S2 | Other *Taxus* species not fire tolerant (Bolsinger and Jaramillo 1990; Sullivan 1993) |
| *Thalictrum thalictroides* |  | E |  | G5, S1 |  |
| *Torreya taxifolia* | X | E | E | G1,S1 | *Torreya californica* fire-intolerant (Howard 1992) |
| *Tipularia discolor* |  | T |  |  |  |
| *Trillium lancifolium* |  | E |  | G3, S2 |  |
| *Veratrum woodii* |  | E |  | G5, S2 |  |
| *Viola hastata var. tripartita* |  | E |  | G5, S1 |  |
| *Woodsia obtusa* |  |  |  | G5, S1 |  |
| *Zanthoxylum americanum* |  | E |  |  |  |
|  |  |  |  |  |  |

Literature Cited in Appendix S2:

Bolsinger, C. L., and A. E. Jaramillo. 1990. *Taxus brevifolia* Nutt. Pacific Yew. Pages 573-579 in: Silvics of North America, Volume 1: Conifers, Burns, R.M., and B.H. Honkala, tech. coords. Agriculture Handbook 654. USDA Forest Service, Washington, D.C

Chafin, L. 2000. Field guide to the rare plants of Florida. Florida Natural Areas Inventory, Tallahassee, FL.

Florida Division of Recreation and Parks 2012. Torreya State Park approved unit management plan. State of Florida, Department of Environmental Protection, Tallahassee, FL.

Florida Natural Areas Inventory (FNAI). 2010. Guide to the natural communities of Florida: 2010 edition. Florida Natural Areas Inventory, Tallahassee, FL.

Howard, J. L. 1992. *Torreya californica*. In: Fire Effects Information System. USDA Forest Service, Rocky Mountain Research Station, Fire Sciences Laboratory.

Nelson, G. and T. Spector. 2011. Protecting endangered plant species in Panhandle State Parks. The Palmetto **28:**4-7.

Spector, T., R. Pruner, J. Cruse-Sanders, M. Friel, J. Smith, R. Determann, and J. Bente. in preparation. Reassessment of the status and condition of the rare conifer Florida torreya (*Torreya taxifolia*) and recommendations for conservation.

Sullivan, J. 1993. *Taxus canadensis*. In: Fire Effects Information System. U.S. Department of Agriculture, Forest Service, Rocky Mountain Research Station, Fire Sciences Laboratory.

US Fish and Wildlife Service (USFWS) 1996. Technical/Agency Draft recovery plan for fringed campion, *Silene polypetala* (Walt.), Fern. & Schub. Atlanta, GA.

Ward, D. B. 1979. Plants, Volume 5 in Rare and Endangered Biota of Florida. University Presses of Florida, Gainesville, FL.
